# Supplementary material for: MetaRibo-Seq measures translation in microbiomes
Source: Nat Commun. 2020 Jun 29;11:3268. doi: 10.1038/s41467-020-17081-z (PMC7324362; doi:10.1038/s41467-020-17081-z)
Supplement: Supplementary file 10 — Supplementary Data 7 [file 41467_2020_17081_MOESM10_ESM.zip › File2/Confidence_VeryHigh_Taxonomy/42259_out.krona.html]

Javascript must be enabled to view this page.

members
magnitude
magnitudeUnassigned
count
unassigned
taxon
rank

42259\_out

5

5
2
superkingdom

2
phylum
976

class
200643
2

2
171549
order

2
171552
family

genus
838
2

1
28132

SRS049147\_contig\_number\_26611
species

species

SRS056323\_contig\_number\_4882
470565
1

phylum
1239
3

class
186801
2

2
186802
order

1898207

SRS1041133\_contig\_number\_13071
species
1

216572
family
1

459786
genus
1


SRS074964\_contig\_number\_contig-100\_7436.7436
species
1945593
1

1
91061
class

186826
order
1

1
family
186828

genus

SRS075033\_contig\_number\_contig-100\_25267.25268
117563
1
